# The aesthetic emotional expression of piano music art in the background of Internet of things

**DOI:** 10.3389/fpsyg.2022.974586

**Published:** 2022-10-12

**Authors:** Xianhua Zhang, Qin Kang

**Affiliations:** ^1^School of Music Education, Shenyang Conservatory of Music, Shenyang, China; ^2^College of Music, Hefei Normal University, Anhui, China

**Keywords:** aesthetic emotion expression, piano music art, musical emotion, Internet of Things, emotional expression

## Abstract

Artwork, generally refers to the work of plastic art. Artwork is divided into many categories, pottery art, traditional Chinese painting, abstract painting, musical instrument, sculpture, cultural relic sculpture, sandstone, imitation sandstone, ornaments, iron art, copper art, stainless steel sculpture and so on. With the continuous influx of artistic works, there are more and more studies on their emotional expression. How to judge whether musical works can bring joy, anger, sadness and joy to people? Is it joy over anger or anger over joy? Now in the era of the Internet of Things, the Internet of Things uses various information sensors, radio frequency identification technology, GPS, infrared sensors, laser scanners and other equipment and technologies to collect any objects and processes that need to be monitored, connected, and interacted in real time. By collecting various information such as sound, light, heat, electricity, mechanics, chemistry, biology, location and so on, and using various possible networks to connect, it can achieve intelligent perception, identification and management of objects and processes. The Internet of Things is an information carrier based on the Internet, traditional telecommunication networks and so on., so that all normal physical objects that can be individually located which can be connected together. The application field of the Internet of Things involves all aspects. The application in the fields of industry, agriculture, environment, transportation, logistics, security and other infrastructure has effectively promoted the intelligent development of these aspects, which making the limited resources more rational use and distribution, thus improving the efficiency and benefit of the industry. The application in household, medical and health, education, finance and service industry, tourism and other fields closely related to life has been greatly improved in terms of service scope, service method and service quality, which has greatly improved people’s quality of life. Based on this, this paper mainly studies the aesthetic emotion expression analysis of piano music art in the context of the Internet of Things. It mainly talks about the classification of music characteristics, emotional theoretical models, and emotional induction methods. Finally, the experimental analysis of piano music and the use of brain wave technology are used to analyze the experimental data. The experimental results show that in the process of feature extraction and optimization, this paper optimizes the traditional feature extraction based on power spectral density through cognitive rules, and achieves the effect of feature dimension reduction on the basis of ensuring the recognition rate. This paper uses the topological properties of EEG to classify emotions. The results show that the emotion recognition rate under the four emotional states can reach 67.3%, which is much higher than the current highest level.

## Introduction

Emotion is a comprehensive state of people, which includes people’s feelings, thoughts and behaviors. The quality of emotions directly affects people’s work and life. Therefore, there are more and more studies on emotions. The analysis and recognition of emotion is an interdisciplinary research involving many fields. It is a characteristic of computers being intelligent to be able to recognize emotions. At present, with the continuous development of artificial intelligence, its research scope has gradually expanded from mechanical intelligence to brain-like. Human emotion recognition is the basis for computers to have emotions, and it is an important trend in the development of artificial intelligence in the future. Emotion computing is a hot research direction at present. Its fundamental purpose is to establish an emotion recognition system with intelligent perception ability, which can make targeted responses according to human emotions (Xun et al., 2022). The concept of affective computing was proposed by Professor Picard of MIT Media Lab in 1997. She pointed out that affective computing is related to emotion, derived from emotion or able to exert influence on emotion. Hu Baogang and others from the Institute of Automation, Chinese Academy of Sciences also put forward the definition of affective computing through their own research and it is that: the purpose of affective computing is to establish a harmonious human-machine environment by giving computers the ability to recognize, understand, express and adapt to human emotions, and to enable computers to have higher and comprehensive intelligence.

Multiculturalism means that the renewal and transformation of culture is also accelerating in the context of more and more complex human society and more and more developed information circulation. The development of various cultures faces different opportunities and challenges, and new cultures emerge one after another. Under the modern complex social structure, it is inevitable to demand various cultures to serve the development of society. These cultures serve the development of society and create cultural pluralism, that is multiculturalism under the complex social background. In recent years, music has played a pivotal role in multiculturalism. Although different cultures have different understandings of music, they all have a common feature, that is, they can make people feel a unique culture in a very short period of time. Music refers to the composer’s use of special tones and melody to convey his thoughts and feelings in different environments. Emotional response to music is the most basic cognitive function of humans. Under different life experiences, different people will have different reactions to the emotions of the same song. And music uses a strong emotion to affect people’s emotions, and in this case, music affects people in the same way. By correctly identifying the emotions of music, it can make harmonious interaction between people, and it has great potential for development in all aspects (Chen, 2018; Chen et al., 2019). For example, in daily life, real-time emotion recognition can adjust one’s emotions in a timely manner, and can effectively use music to affect people’s emotions, thereby reducing unexpected events caused by emotions. In the medical field, if the laws of people’s emotional cognition can be understood, it will have a certain guiding role for the treatment of emotional disorders.

This paper mainly talks about the classification of music features, emotional theoretical models, emotion induction methods, and feature extraction of music emotions. Then it also conducts experimental analysis on piano music, and designs experimental data of four emotional dimensions. Finally, the brainwave technology is used to analyze the experimental data, which also leads to the final experimental results of this paper achieving the highest accuracy in history. The innovation of this paper lies in the perfect use of current high-tech technology, and the experimental data is also analyzed layer by layer, and the final result is also very accurate and interlocking.

## Related work

In today’s era of artistic works in full bloom, aesthetic emotions are regarded as various emotional responses to works of art. The piano art, in particular, originated and formed from the late 1920s–1930s, developed from the 1940s–1960s, and reached its heyday from the 1980s–1990s. Revenko (2019) analyzed the best examples of this genre in the works of domestic composers past and present. It turned out that the positive qualities of L. Revutsky’s Second Piano Concerto lied in the organic development of the Ukrainian classical tradition, in the profound manifestation of a noble, cheerful mood, lyrically excited sadness and strong-willed image. It has recently been suggested that more cognitive and complex tactile processes, such as music perception, could help uncover the superior tactile abilities of deaf people. Indeed, deaf music seems to be perceived through vibrations. But the extent to which they perceive musical characteristics through tactile means remains undetermined. The purpose of Sharp A was to investigate the tactile recognition of musical emotion in deaf people. Participants had to rate melodies based on their emotional perception (Sharp et al., 2020). Timbre is an important factor affecting emotional perception in music. To date, little is known about the impact of timbre on emotional neural responses to music. To address this issue, Zhang et al. (2019) used ERP to investigate whether there are different neural responses to musical emotion when the same melody is presented in different timbres. There is very little academic literature on conceptual fusion and emotion. Thus, Spitzer M reconciled the cognitive metaphor of anger with conceptual mixing theory, which enabling the article to analyze the two parts of anger in Vivaldi and Haydn separately. This analysis raised some relief problems when applying conceptual fusion to aesthetic objects in general, and musical compositions in particular (Spitzer, 2018). They all discussed and analyzed the piano art to varying degrees, but they were not innovative enough, the research value was not enough, and they did not invest more emotion.

Musical stimulation can induce emotions, and can also regulate and improve emotions. However, for the scientific experimental research on the improvement of music-induced emotion and music-regulated emotion, it is not enough to prove it through scientific experiments, and more importantly, it needs further explanation and confirmation. By further studying the influence and neural mechanism of musical stimulation on the brain, and how this influence affects the physiological function system of the brain, the physiological function system of the human brain can explained. The distinction between “music-induced emotion” and “musical emotion,” as well as between the “referentialist” and “absolutist” (or “cognitivist”) schools of music psychology constitutes the question. Kramarz (2017) explored how far the concept of “musical emotion,” a term coined by contemporary psychology, dated back to antiquity. While most ancient theorists believed that the influence of music on passion was of pedagogical or therapeutic relevance, it was only because of its ability to create ethos in the human soul through imitation. But others, like the cognitivists, limited their influence to (aesthetic) pleasure (Kramarz, 2017). Sutcliffe R investigated differences in emotion recognition in young adults using music and facial stimuli, and tested explanatory hypotheses about the generally poorer emotion recognition in older adults. In the experiments, young and old were labeled emotions in a given set of faces and classical piano stimuli. And this paper was pilot tested on other young and older adults. Older people are even less good at spotting anger, sadness, fear, and joy in music (Sutcliffe et al., 2018). The possible functional changes and possible outcomes under the stimulation of music can be used to study the role of music in clinical treatment and its possible effects on neuromedical interventions of music. Wu and Sun (2018) selected pieces of four emotions, “happy,” “sad,” “soothing” and “stressful,” and took “soothing,” “happy” and “stressful” as the main components. At the same time, the EEG signal was recorded, and the EEG signal was detected. They all explained and contrasted the various emotions of the music. However, in the experiments they conducted, the data analysis was not accurate enough, and they did not conduct in-depth research using techniques such as brain waves. Therefore, the conclusions drawn are not accurate enough.

## Aesthetic emotion expression algorithm for music art

### Affect theory model

**FIGURE 1:**
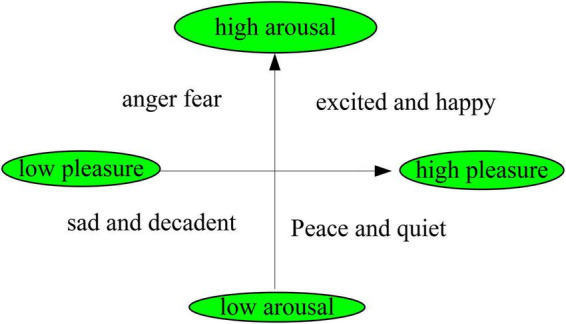
Dimensional theoretical model of emotion.

Emotion research has a long history, and scholars have explored the generation and development of emotion from multiple perspectives. The first one of the basic affect theory is a basic affect theory. It includes basic emotions, which including basic positive emotions such as excitement, joy, happiness, sadness, anger, disgust, fear and so on. The second one is the theory of emotional dimension. The basic idea of this theory is that people’s core emotions persist in the brain, and it contains two levels of pleasure (unpleasant-pleasure) and arousal (unawakened-arousal). Its expression is shown in Figure 1. This theory has been accepted by most scholars.

At present, there are three common ways of emotional induction:

(1) Through external stimuli such as sounds and images, the subjects can produce various emotions. This method induces the subjects to generate corresponding emotions by allowing the subjects to hear emotional sounds or watch emotional videos, so as to obtain stable and precise emotions, and is currently the main emotion induction method.
(2) Emotions are stimulated by using the subject’s facial expressions. This method induces emotions by prompting and inducing subjects to make facial expressions that correspond to emotions. But the study couldn’t determine whether the subjects’ facial expressions matched their real emotional state.
(3) Emotions are stimulated by using memories. This method is only based on the subject’s intrinsic response, and when the subject lacks specific emotional memory, the corresponding emotion cannot be generated (Ferran, 2018; Lee et al., 2020). At the same time, the emotions elicited by this method cannot ensure that the subjects can recall their own emotions.

### Emotional characteristics of music

#### Acoustic features

##### Short-term zero-crossing rate

The short-term zero-crossing rate is a characteristic of the signal time domain. If the algebraic signs of the first and last two sample points in the audio time-domain discrete signal are inverted, it is called a zero-crossing. The rate of zero-crossing is called the zero-crossing rate, which reflects the spectral characteristics of the signal to some extent. Usually a high zero-crossing rate means that the signal is concentrated in the high frequency part (Qian et al., 2019). A low zero-crossing rate means that the signal is concentrated in the low frequency band. The specific calculation formula is:


(1) $$w_{d}=\frac{1}{2\,\left(M-1\right)}\,\sum_{n=1}^{M-1}\left|s\,g\,n\,\left[a\,\left(n+1\right)\right]-s\,g\,n\,\left[a\,\left(n\right)\right]\right|$$

_*a(n)*_ is the audio time-domain discrete signal, m is the sampling times of a frame signal, and sgn[] is the sign function.

##### Spectral centroid

The spectral centroid is an index to measure the spectral shape and spectral brightness. It is the center of the amplitude spectrum after short-time Fourier transform of the audio signal, and also called brightness. The larger the spectral centroid value is, the higher the quality of the signal and the higher its frequency are. The specific calculation formula is:


(2) $$N_{d}\,\left(m\right)=\sqrt{\sum_{j=1}^{r}Q_{d}^{2}\,\left(m\right)\,/\,r},U_{d}=\frac{\sum_{m=1}^{M}N_{d}\,\left(m\right)*m}{\sum_{m=1}^{M}N_{d}\,\left(m\right)}$$

Among them, the Fourier transform magnitude represents the d-th frame of the m-th window.

##### Spectral energy value

It is also called spectral attenuation, which refers to the cutoff frequency corresponding to 95% of the total energy of the spectrum and is used to describe the shape of the spectrum. Its formula is:


(3) $$t_{j}=\sum_{i=1}^{M_{i}}P\,\left[S_{j}\,\left(i\right)\right]\leq 0.95\cdot \sum P\,\left[S_{i}\,\left(n\right)\right]$$

Among them, *t*_*j*_ is the energy accumulated before the cut-off frequency of the signal in one frame, and more than 95% of the total energy will not be accumulated (Boonipat et al., 2021).

##### Short-term energy

The short-term energy is used to reflect the change trend of the amplitude of the speech signal, and it is mainly used to distinguish the clear and voiced parts. Its formula is:


(4) $$T_{m}=\frac{1}{M}\,j\,\sum_{i=n}^{M-1}{\left[P_{m\,\left(j\right)}\,q\,\left(m-n\right)\right]}^{2},q_{\left(j\right)}=\left\{ \begin{array}{c} 1,0\leq j\leq M-1\\ 0,o\,t\,h\,e\,r\,w\,i\,s\,e \end{array}\right.$$

It is generally used to take the logarithm of it.

##### Mel Cepstral Coefficients

This paper believes that if the human cochlea is regarded as a filter, then its filtering function is on the logarithmic level. Below 1000 Hz, the perceptual ability of the human ear is linearly related to frequency. Above 1000 Hz, human hearing ability is logarithmic with frequency, which makes the human ear more than logarithmic to low-frequency signals. Mel-Frequency Cepstral Coefficients (MFCCs) are the coefficients that make up the Mel-Frequency Cepstral. It is derived from the cepstrum of an audio clip. The difference between cepstrum and mel-frequency cepstrum is that the frequency band division of mel-frequency cepstrum is divided equally on the mel scale. It more closely approximates the human auditory system than the linearly spaced frequency bands used in the normal cepstrum. Such non-linear representations can lead to better representation of sound signals in various fields, such as in audio compression. MFCC builds a human ear model based on this feature, and has been widely used in music recognition and signal processing (Kim, 2017).


(5) Mel(p)=2595lg(1+p/700

The transformed spectrum is called the Mel cepstral region. The Mel cepstral coefficient is a set of parameters that can reflect the emotional characteristics of music obtained by converting the audio signal to the cepstral region.

##### Linear Prediction Coefficients

Linear prediction is a method of predicting the estimated value of the current sample value Sn according to the sequence of p known sample values of the random signal in the past as Sn-1, Sn-2, … Sn-p. The prediction formula is a linear equation, so this kind of prediction is called linear prediction. The linear prediction model mainly uses the all-pole mode to simulate the speech signal, and regards the speech signal x(n) as the output of the slowly changing vocal tract system under the excitation of the glottal u(n). The expression of the voice signal is:


(6) $$a\,\left(m\right)=\sum_{j=1}^{M}x_{j}\,a\,\left(m-j\right)+S\,w\,\left(m\right)$$

_*x_j_ (j=1,2 …M)*_ is the filter coefficient and M is the filter order. When the audio signal is voiced, w(m) is a pulse signal of one period. And in the unvoiced case, w(m) is a random noise sequence and S is the gain of a filter. By finding the coefficients of the filter, a linear prediction model can be obtained by:


(7) Tm=∑j=m-M+fm(Q-j∑i=1fxiQj-i)2

#### Perceptual features

##### Subband spectrum average peak or average valley difference

According to the 7 sub-spectral vectors contained in the spectrum of each frame, $\left\{a_{1}^{\prime },a_{2}^{\prime }\,\ldots \,a_{m}^{\prime }\right\}$ is arranged according to its size. In a closed interval, the average value of the maximum and minimum values are:


(8) $$F_{e\,a\,k}=\left\{\frac{1}{\beta \,M}\,\sum_{j=1}^{\beta \,M}a_{r,j}^{\prime }\right\}$$

(9) $$L_{a\,l\,l\,e\,y}=\left\{\frac{1}{\beta \,M}\,\sum_{j=1}^{\beta \,M}a_{r,M-j+1}\right\}$$

The difference is took between Formulas (8) and (9):


(10) $$H=F_{r}-L_{r}$$

##### Subband amplitude envelope

The Hamming window (Stamatopoulou and Cupchik, 2017) is used to convolve the spectral vectors of 7 subbands in a frame, and the obtained values represent the amplitude envelope characteristics of each subband. Its formula is:


(11) $$X_{j}^{\prime }\,\left(r\right)=X_{j}\,\left(r\right)⊗g_{z}\,\left(m\right)$$

The Hamming window is:


(12) $$g_{z}\,\left(m\right)=0.5+0.5\,\cos\left(2\,\pi \,\frac{m}{2\,V-1}\right),m\in \left[0,V-1\right]$$

The subband magnitude envelope is:


(13) $$Q_{j}=\sum_{j=1}^{7}X_{j}^{\prime }\,\left(r\right)$$

##### The peak, valley and difference of melody change

The sub-band amplitude envelope is smoothed by a Gaussian filter. In order to detect the rate of change of the envelope, the method of first derivation and then autocorrelation is adopted. Its calculation formula is:

The Gaussian filter is:


(14) $$P_{j}^{\prime }m)=P{}_{j}(m)⊗\{\frac{m}{\varepsilon^{2}}u^{-\frac{m}{\varepsilon^{2}}}\},m\in [-V,V]$$

In the Formula, V and ε are Gaussian filter parameters.

### Feature extraction

#### Short-term feature extraction

Music feature extraction is the foundation of content-based music classification systems. The selection of appropriate features is crucial to the performance improvement of the classification system. Audio content features can be divided into three levels according to the degree of abstraction. The bottom layer is the physical sample layer, the middle layer is the acoustic feature layer, and the top layer is the semantic layer. The higher the level is, the abstraction of the content is higher.

##### Pre-emphasis

A digital filter is an algorithm or device composed of digital multipliers, adders and delay units. The function of the digital filter is to perform arithmetic processing on the digital code of the input discrete signal to achieve the purpose of changing the signal spectrum. Before feature extraction, the music signal should be preprocessed. The preprocessing work mainly includes digitization, pre-emphasis, windowing and framing. The music signal used in this paper takes the common wav format on the Internet, and no digitization operation is required (Babacan and Babacan, 2017). In order to facilitate the analysis of spectrum and channel parameters, a high-pass digital filter is used to process the digital signal, and the following Formula is obtained:

The digital filter is:


(15) $$G\,\left(w\right)=1-\varphi \,w^{-1}$$

Among them, 1 is the pre-emphasis coefficient, and the general size is between 0.9 and 1.0.

##### MFCC feature extraction

**FIGURE 2:**
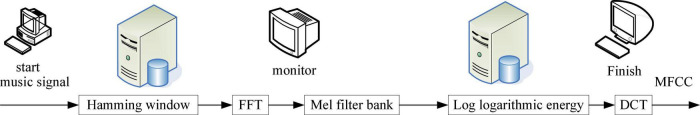
MFCC feature extraction process.

The extraction process of MFCC is shown in Figure 2:

#### Hamming window

Hamming window is an electronic term promulgated by the National Committee for the Approval of Scientific and Technological Terms in 1993. It was published in 1993 and was approved and released by the National Committee for the Approval of Scientific and Technological Terms. And it is from the first edition of “Electronics Terms.” After pre-emphasizing the music signal, it needs to be windowed and divided into frames to effectively extract its short-term features. Due to the short time-lag stability of the music signal, it is generally considered that between 10 and 30 ms, the characteristics of the music segment signal can be regarded as a frame signal (Manno et al., 2019). The method of windowing and framing is to use a movable window of limited length (commonly used rectangular window, Hanning window) to intercept the music signal, and use the characteristics of a frame of signal to characterize its short-term characteristics. The expression is:


(16) $$a\,\left(m\right)=\sum_{-\infty }^{+\infty }D\,\left[P\,\left(n\right)*u\,\left(m-n\right)\right]$$

Among them, *a*(*m*) is the intercepted signal, *D*[⋅] is a certain type of transformation, p(n) is the pre-emphasized signal, and u(m-n) is the window function. The window function used in this paper is the Hamming window, and its expression is:


(17) $$u\,\left(m\right)=\left\{ \begin{array}{cc} 0.54-0.46\,\cos\left(2\,\pi \,/\,M-1\right), & 0\leq m\leq M-1\\ 0, & e\,l\,s\,e \end{array}\right.$$

The sampling rate of the music signal in this paper is 16 Khz, the frame length is 32 ms, and the frame shift is 16 ms. Therefore, the number of sampling points per frame is *N* = 512, and the number of sampling points for frame shift is 256.

#### Fast fourier transform

Fourier transform means that a function that satisfies certain conditions can be expressed as a trigonometric function (sine and/or cosine function) or a linear combination of their integrals. In different research fields, Fourier transform has many different variants, such as continuous Fourier transform and discrete Fourier transform. Fourier analysis was originally proposed as a tool for analytical analysis of thermal processes. By performing fast Fourier transform (FFT) on a frame of music signal *a*(*m*) obtained above, a linear spectrum *A*(*r*) is obtained, and the calculation formula is:


(18) $$A\,\left(r\right)=\sum_{m=0}^{M-1}a\,\left(m\right)\,U^{m\,r},r=0,1,2,\ldots \,M-1$$

#### Design of Mel filter bank

**FIGURE 3:**
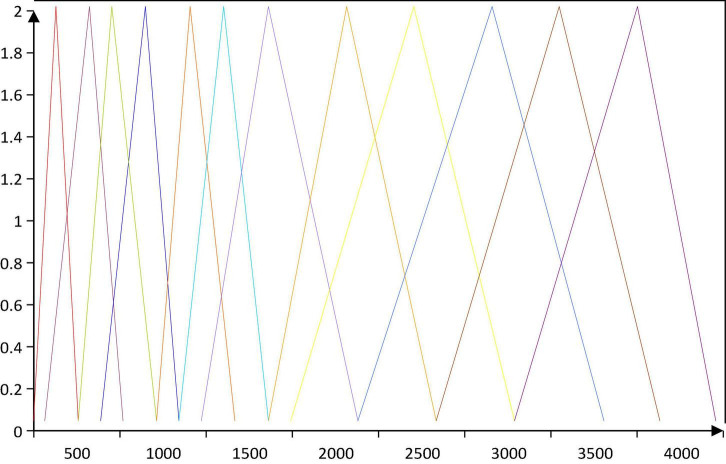
Triangular filter distribution in real frequency domain.

The Mel filter bank consists of triangular bandpass filters with equally spaced center frequencies g(n) in the Mel frequency domain, and increases with n in the real frequency domain, as shown in Figure 3:

The sampling rate of the music signal in this paper is 16 Khz, then *g*_*max*_ = 8 Khz, and the center frequency interval of each triangular filter on the Mel domain is: $\Delta \,M\,e\,l=\frac{g_{\max}}{r+1}$. The transfer function *Z*_*n*_(*r*) of the Mel filter bank is defined as:


(19) $$Z_{n}\,\left(r\right)=\left\{ \begin{array}{cc} 0, & e\,l\,s\,e\\ \frac{r-g\,\left(n-1\right)}{g(n-g\left(n-1\right)}, & f\,\left(n\right)\leq r\leq f \end{array}\right.$$

g(n) is the center frequency of the nth triangular filter, and the calculation formula is:


(20) $$g\,\left(n\right)=\left(\frac{M}{G_{w}}\right)\,Y^{-1}\,\left(Y\,\left(g_{1}+n\,\frac{Y\,\left(g_{e}\right)-Y\,\left(g_{1}\right)}{N+1}\right)\right)$$

Among them, *g*_1_*g*_*e*_ is the maximum and minimum frequencies of the triangular filter, N is the number of sampling points, *G*_*w*_ is the sampling rate of the music signal, and *Y*^−1^(⋅)is the conversion formula from Mel frequency to real frequency:


(21) $$Y^{-1}\,\left(y\right)=700\cdot \left(v^{y\,/\,2595}-1\right)$$

## Experimental design of brain cognition of music emotion

### Experimental sampling

**FIGURE 4:**
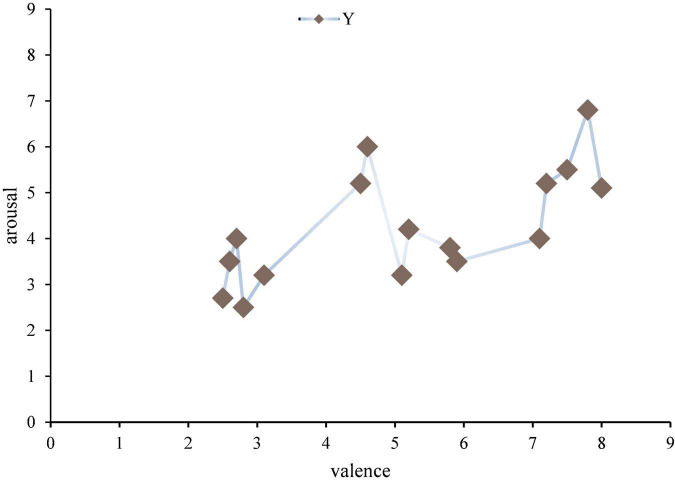
Music emotion distribution map.

The excitation material is 16 pianos, each 30 s, the volume is within 60 decibels of the human ear can hear the comfort level. According to: a total of 50 piano works, 10 volunteers who did not participate in the experiment filled in the emotional scale in order, and after analyzing all of them, 16 works with the greatest emotional fluctuation were selected, as shown in Figure 4.

Emotional pattern refers to a system in which each specific emotion has its unique neurophysiological basis and experiential characteristics. Each emotion archetype corresponds to the original adaptive mode and its associated behavioral mode. For example, American psychologist Placek believes that the following eight emotional archetypes have their corresponding adaptation and behavior patterns. Different emotional experience patterns produced by different combinations of different emotions in intensity. The results show that the distribution of the samples in the second quadrant is small. From Lange’s two-dimensional emotional model, the second dimension is emotions with a lower degree of happiness and a higher degree of arousal, mainly anger, tension and other emotions. This is consistent with previous studies and the material chosen for this paper. Because music is more difficult to generate negative emotions such as anger, or even not strong. This paper will not have any effect on the mental health of the subjects when choosing stimulants, and in this quadrant, the choice of stimulants is also very small and has no effect on the mental health of the subjects. The subjects participating in the trial were 21–23 years old (27 males and 5 females). All subjects are right-hand grips, and the subjects’ native language is Chinese, and the second language is English; all patients have no neurological disorders, and no relevant genetic history; subjects do not have any professional music background.

### Experimental process

EEG data is obtained. Firstly, the purpose of the experiment, the process and the problems that need to be paid attention to are introduced. The subjects filled out the personal data form, made a commitment, and signed the consent to the trial report. Then, on the subject’s brain wave acquisition device, the subject sits in a comfortable seat with eyes fixed on the center of the display screen, and the distance between the eyes and the screen is 60–80 cm. While the music is playing, a “+” number appears in the middle of the screen, and subjects are required to fill out a mood quantification form after listening to the music. This quantification table includes happiness and arousal. From 1 to 9, it represents happiness or arousal. And subjects need to fill in according to their actual situation. After the experiment, the EEG acquisition device of the experimental subjects was taken off, cleaned, and given material rewards.

The whole experimental procedure is shown below. The experiment is divided into 16 trials, and each trail consists of the following parts:

(1) After 15 s of rest, EEG data were collected.
(2) Music was played and EEG data were collected for 30 s.
(3) After 15 s of rest, EEG data were collected.
(4) The subjects filled out an emotion scale.
(5) It was rest time for 30 s.

### Data collection

#### Acquisition equipment

**FIGURE 5:**
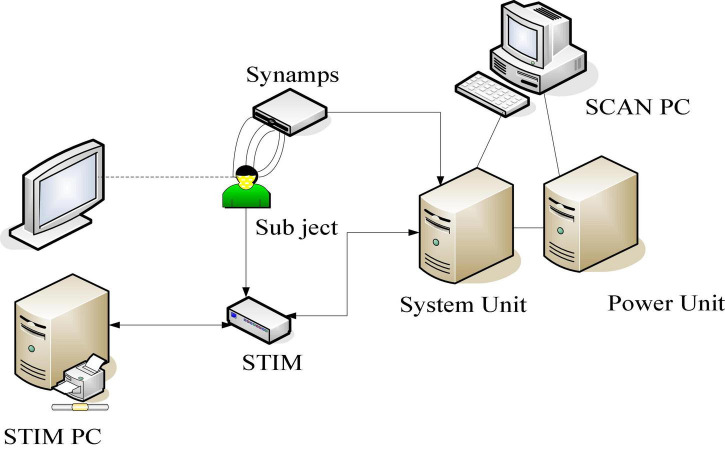
Schematic diagram of EEG data acquisition equipment.

In this experiment, the replay system of Presentation software was used, and the corresponding tests were carried out according to the above experimental process. The experiments were all carried out in a quiet room set up in the laboratory, and the lighting conditions were always controlled during the experiment. The EEG signal was recorded by NeuroScan ECG, and the EEG signal was recorded by Scan4.5 (NeuroScan Inc., Herndon, United States) of United States (NeuroScan Inc., Herndon, United States), and all signals were amplified by SynAmp2, and then sent it to the main control computer for recording. The acquisition equipment is shown in Figure 5.

A total of 10–20 System electrode naming rules are: each electrode is named with the English abbreviation of the brain region as the head. Electrodes in the left hemisphere are named with odd numbers, while those in the right hemisphere are named with even numbers; electrodes near the midline are named with smaller numbers, and electrodes closer to the lateral side are named with larger numbers.

To remove EEG noise, two electrodes are placed near the eyes to collect vertical eye movement artifact and horizontal eye movement artifact, which are used for eye movement artifact removal during EEG signal preprocessing. This device uses high-density leads in order to increase the accuracy of source localization. In order to accurately obtain the potential difference between the leads, a reference electrode needs to be set. The potential of the ideal reference electrode point is set to zero. Electrodes other than the reference electrode are also collectively referred to as recording electrodes. The reference electrodes in this study were set at the mastoid of both ears, and the EEG at the mastoid is generally smaller and more in line with the needs of EEG signal measurement. By using the average potential of two points as a reference, the distortion of the potential information of the two hemispheres of the brain is not caused.

#### Data composition

Each EEG data consists of 16 segments of data in the same format, each segment mainly contains three parts of EEG data: the resting state of EEG signal before listening to music (ideally, the subject has no emotional activity at this time, no intense brain activity, and is in a calm state), EEG signals under long-term musical stimulation (ideally, this EEG data includes the brain’s perception of music, the brain response to changes in music characteristics, the generation of emotions and so on), EEG signals that re-enter the resting state after listening to music (ideally, brain activity in this state should transition from an active state stimulated by music to a resting state) (D’Onofrio et al., 2018, 2019).

**FIGURE 6:**
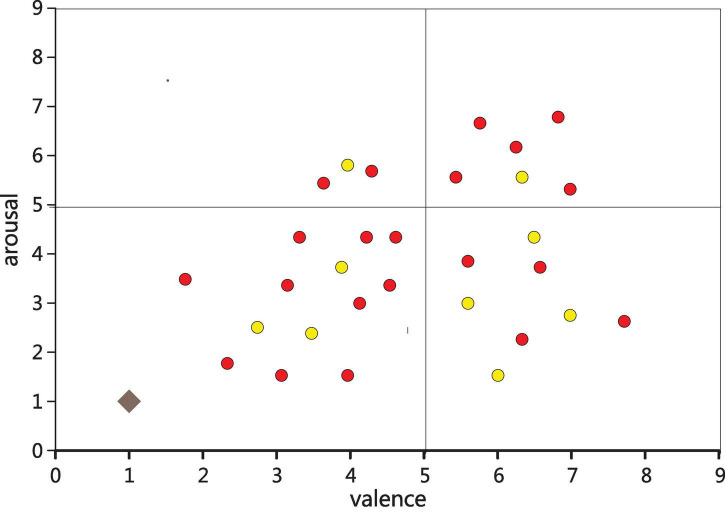
Music sentiment distribution map in the database.

This paper firstly collects the questionnaires of the subjects’ emotional state, obtains their emotional state when listening to music, then counts their emotional state, and finally obtains the statistical distribution as shown in Figure 6.

### Data resolve

**FIGURE 7:**
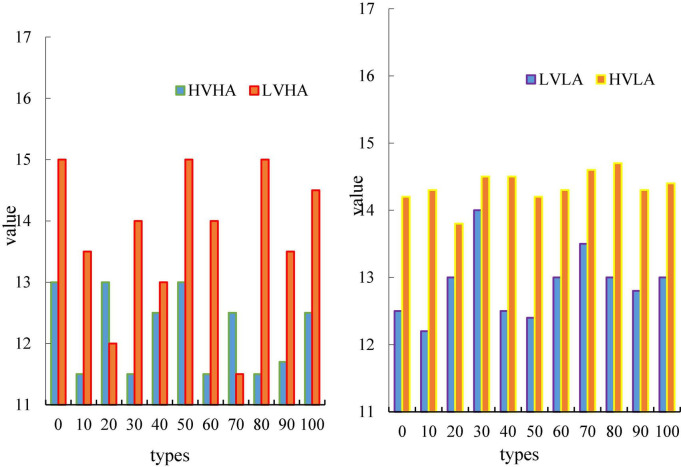
Time graph of average node degree.

In this chapter, this paper divides pleasure and arousal into high pleasure and high arousal (HVHA), high pleasure and low arousal (HVLA), low pleasure and high arousal (LVHA), low pleasure and low arousal (LVLA). Figure 7 is a brain network adjacency matrix for four different emotion types in the delta band during 0–500 ms.

The results shown in Figure 7 are the average of all subjects in this emotion category (Kerkova, 2018). It can be seen from this figure that the adjacency matrix values on the diagonal are generally relatively high. This suggests that each electrode in the EEG contains information about multiple adjacent electrodes. On the adjacency matrix diagram, it can be clearly seen that the mutual information between this aggregation and several adjacent electrodes is high.

Figure 7 is a graph showing the average nodularity of the four emotions as a function of time, wherein the horizontal axis represents time, and the vertical axis represents nodularity. In this paper, it can be clearly seen from the Figure that the two types of emotions, HVLA emotion and HVHA emotion, have a clear degree of distinction. From the beginning to the end, there is no intersection, and the average node degree of HVLA is significantly higher than that of HVHA (Durahim et al., 2018), that is, under high pleasure, there is a clear distinction between the level of arousal. Similarly, LVHA emotion and LVLA emotion, the distinction between the two is also more obvious. The figure shows that under the condition of a certain degree of pleasure, the difference in arousal is the main factor that causes the difference in the amplitude of the nodes in the brain network.

**FIGURE 8:**
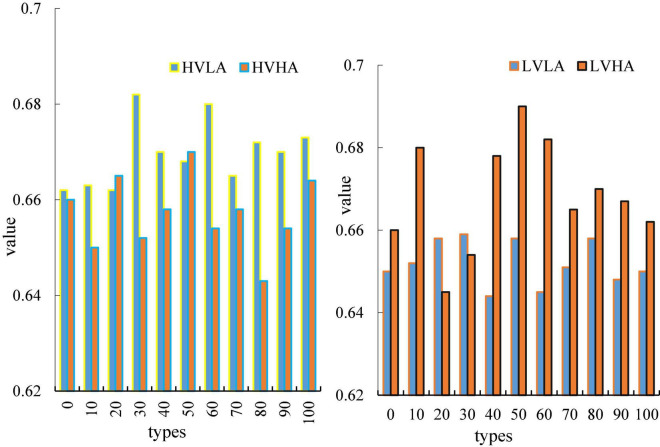
Time variation plot of average clustering coefficient.

Figure 8 is a curve of the average clustering coefficient of the four emotions over time, wherein the abscissa represents time and the ordinate represents nodes. The average clustering coefficient of different emotions has a similar law to the average node degree. In this paper, it can be clearly seen from the Figure that the two types of emotions, HVLA emotion and HVHA emotion, have a clear degree of distinction. From the beginning to the end, there is no intersection, and the average node degree of HVLA is significantly higher than that of HVHA (Durahim et al., 2018), that is, under high pleasure, there is a clear distinction between the level of arousal. Similarly, LVHA emotion and LVLA emotion, the distinction between the two is also more obvious. The figure shows that under the condition of a certain degree of pleasure, differences in average clustering coefficients of brain networks can be used to distinguish differences in arousal.

**FIGURE 9:**
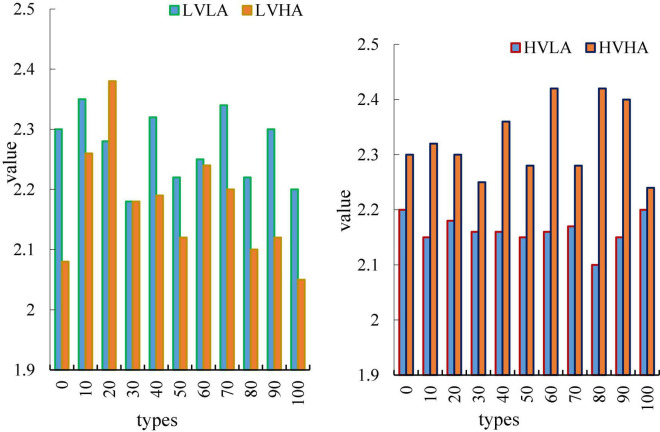
Time variation plot of average path length.

Figure 9 is a graph of the average route length of the four emotions as a function of time, where the horizontal axis represents time and the vertical axis represents node degrees. In this paper, it can be clearly seen from the Figure that the two types of emotions, HVLA emotion and HVHA emotion, have a clear degree of distinction. From the beginning to the end, there is no intersection, and the average node degree of HVLA is significantly lower than that of HVHA. This is the opposite of the average clustering coefficient, which explains the dynamic balance of the brain. When the average clustering coefficient increases, it indicates that there is frequent information exchange between some brain regions in the brain and surrounding brain regions. At this time, by reducing the average path length of brain regions, the efficiency of information interaction between brain regions can be improved. This partly explains the self-regulation of the brain. Similarly, LVHA emotion and LVLA emotion, the distinction between the two is also more obvious (Grace et al., 2019).

This section also uses two datasets. The distribution of samples in the two datasets is shown in Table 1.

**TABLE 1:** Sample distribution.

	HVHA	HVLA	LVHA	LVLA
Dataset 1	128	64	160	160
Dataset 2	192	256	448	384
Difference	Y	Y	Y	Y

Classification is a very important method of data mining. The concept of classification is to learn a classification function or construct a classification model based on the existing data (that is, the so-called classifier). The function or model can map the data records in the database to one of the given categories, which can be applied to data prediction. In a word, classifier is a general term for the methods of classifying samples in data mining, including decision tree, logistic regression, naive Bayes, neural network, and other algorithms. This paper first uses the extracted brain network features for emotion recognition. In emotion recognition, three classification tasks are set up in this paper, namely the two-classification task based on arousal, the two-classification task based on pleasure, and the four-classification task based on arousal. This article uses a variety of classifiers such as MLP, SVM, KNN, baggedtree and so on. The recognition results of the binary classification task based on high and low arousal are shown in Table 2, and the recognition results of the binary classification task based on pleasure are shown in Table 3.

**TABLE 2:** Binary classification emotion recognition results based on arousal.

	MLP	SVM	KNN	Bagged tree
Node degree	71.1%	72.5%	61.7%	71.1%
Clustering coefficient	71.1%	71.1%	67.1%	70.4%
Draw path	71.1%	72.5%	65.4%	71.7%
ALL	73.8%	73.8%	66.3%	71.3%

**TABLE 3:** Binary classification emotion recognition results based on pleasure.

	MLP	SVM	KNN	Bagged tree
Node degree	70.8%	70.8%	64.6%	66.7%
Clustering coefficient	70.8%	70.8%	52.9%	67.5%
Draw path	70.8%	70.8%	65.8%	66.7%
ALL	70.8%	70.8%	68.3%	67.1%

**FIGURE 10:**
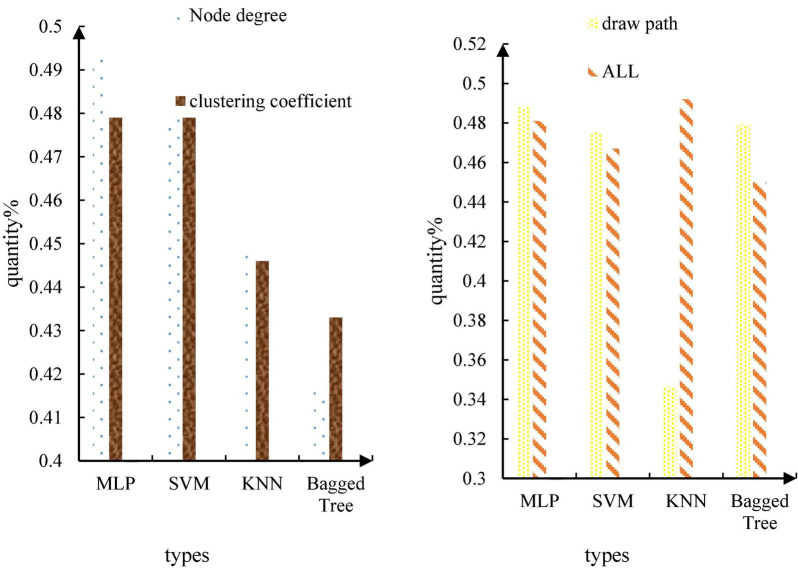
Recognition results of the four-classification task based on pleasure arousal.

The recognition results of the four-classification task based on pleasure arousal are shown in Figure 10.

The results show that the recognition rate of the binary task of emotion classification based on the characteristics of the brain network is 73.8%, which is obviously better than the emotion recognition based on the time-frequency characteristics. This shows that the brain dynamic network in this paper can effectively distinguish different emotions, and the emotion recognition based on alertness is much better than the emotion recognition based on pleasure. This medical research has crossed into the military field. The United States Department of Defense has invested $24 million in various “mind-controlled robots” research. The ultimate goal of this plan is to create a “machine warrior” or a new type of unmanned aircraft that can be completely controlled by mind. Brain network properties were more discriminative for differences in arousal. From the classifier, it can be seen from this paper that the performance of MLP and SVM is the best, and the recognition rates of the two are almost the same. The feature used above is extracted through the brain network, which is a high-order feature, because the brain network itself is also a topological feature of the EEG signal. Therefore, this paper considers directly using the brain network itself as a feature and feeding it into the classifier for emotion recognition. There are 32 subjects in the self-collected database, each subject has 16 songs, each song is 30 s, and the EEG data sampling rate after preprocessing is 1000 Hz. In this paper, the 30 s-long EEG data is divided into a 300 ms segment of EEG data, and a brain network of 4 frequency bands is made for each segment of EEG data. The adjacency matrix formed by the brain network of 4 frequency bands is used as the input of MLP. After segmenting the EEG signals, dataset 1 has a total of 32*16*100 = 51200 samples. The fivefold cross-validation method is adopted, and 80% of the samples are taken for training each time, and 20% of the samples are used for testing. The recognition results are shown in Table 4.

**TABLE 4:** Sentiment classification results based on adjacency matrix.

	Arousal	Pleasure	Four categories
MLP	78.5%	75.1%	67.3%
SVM	78.5%	74.5%	65.2%
Difference	N	Y	Y

According to the experimental results, this paper finds that it is better to use the adjacency matrix of the brain network directly as a feature for emotion recognition. Under the four classifications, the recognition rate of 67.3% is achieved, which is the best result for music emotion at present.

## Conclusion

First, based on long-term music appreciation, an emotional induction experiment IAS conducted. On this basis, brain wave detection is performed on 32 subjects.

Then, on the basis of the previous research results, the traditional feature extraction method based on energy spectrum is improved, and the cognitive rules are used to optimize it, so as to realize the feature dimensionality reduction under the premise of maintaining the recognition rate. This study utilizes the topological properties of EEG to classify emotions. The results show that the emotion recognition rate under the four emotional states can reach 67.3%, which is much higher than the current highest level.

Therefore, this paper conducts research from two aspects. First, this paper examines the emotional changes evoked by the brain during music processing. By studying the neural mechanism of emotion induction, it can provide a neuroscience basis for this paper to better identify emotional states. In addition, this paper also analyzes and classifies the behavioral characteristics of the musical emotional brain. The innovative results of this subject mainly focus on the music event points and dynamic brain network, and have achieved good results. In the future, the improved work of this part will be continued to make up for the current deficiencies, and hope to achieve good results.

## Author’s note

XZ was born in Fushun, Liaoning, China in 1978. She obtained a master’s degree in piano performance from Shenyang Conservatory of Music. She is currently a piano teacher at Shenyang Conservatory of Music. Her research interests include piano performance and piano pedagogy. E-mail: zhangxianhua202203@163.com; QK was born in Wuhu, Anhui. China in 1980. She received the Master degree in musicology from the Russian State Normal University. She is an associate professor of musicology in College of Music of Hefei Normal University. Her research interests include music analysis, piano education, and aesthetics of traditional Chinese music. E-mail: kangqin316@163.com.

## Data availability statement

The original contributions presented in this study are included in the article/supplementary material, further inquiries can be directed to the corresponding author.

## Author contributions

XZ: writing – original draft preparation. QK: editing data curation and supervision. Both authors contributed to the article and approved the submitted version.
